# Complete plastome genome of *Pergularia tomentosa* L. (Asclepiadoideae, Apocynaceae)

**DOI:** 10.1080/23802359.2019.1710291

**Published:** 2020-02-06

**Authors:** Abidina Abba, Dhafer Alzahrani, Samaila Yaradua, Enas Bokhari Albokhari

**Affiliations:** a Faculty of Science, Deparment of Biological Sciences, King Abdulazeez University, Jeddah, Saudi Arabia;; b Faculty of Science, Deparment of Biological Sciences, Federal University, Lokoja, Nigeria;; c Faculty of Applied Sciences, Department of Biological Sciences, Umm Al-Qura University, Makkah, Saudi Arabia

**Keywords:** Asclepiadeae, conservation, evolution, phylogeny

## Abstract

*Pergularia tomentosa* is a medicinal plant mainly found in Saudi Arabia, northern and southern Africa. In this study, we present the sequence of the complete chloroplast (cp) genome of *P. tomentosa* in order to evaluate the evolutionary relationship in the subfamily Asclepiadoideae. The cp is 164,213 bp in lengh with 37.3% GC content, inverted repeat (IR) regions of 21,411 bp each, a large single-copy (LSC) region of 80,102 bp, and a small single-copy (SSC) region of 17,022 bp. It constitutes of 89 protien-coding genes, 44 tRNA genes, and 8 rRNA genes. The phylogenetic relationship showed close relationship between *P. tomentosa* and other Asclepiadeae members with Marsedineae subtribe. The study will help for future research on evolutionary studies of Apoceanaceae.

## Introduction

*Pergularia tomentosa* L. is a perennial plant belonging to Apoceanaceae, it has a unique hairy surface and greenish color. *Pergulria* is a member of subtribe Asclepiadinae, alongside *Asclepias*, *Calotropis*, and *Garmphocarpus*, with two major polymorphic species mainly *Pergularia daemia* and *P. tomentosa*. It is found in the desert and dry areas of southern Africa up to the northern parts of the Arabian Peninsula. Conservation of this plant is very important considering its potential in ethnomedicine and the effects of climate change on Biodiversity.

The chloroplast (cp) is an important part of plant cells and a major distinguishing feature that separates plants from animals and is very useful for life on Earth (Jin and Daniell 2015). We present the assembled sequence of the complete cp genome of *P. tomentosa* (Genbank accession number: MN548766) for the purpose of providing bases of genetic information for future research.

Fresh leaves of *P. tomentosa* were collected from Al-Shafa mountains (22°4.8′32′′N, 40°18.6′28′′E), 21 km from the Al-Taif city, Kingdom of Saudi Arabia. The voucher specimen was deposited in the herbarium of the King Abdulazeez University with the assigned number KAU27331. DNA extraction was done using the Qiagen DNA extraction kit (Seoul, South Korea) based on the manufacturer’s guidelines. The Extracted DNA was sequenced using Illumina Hiseq 2500 platform (Novogene Technology, Inc., Beijing, China). Raw data were filtered using PRINSEQ lite Ver 0.20.4 (http://prinseqsourceforge.net; Schmieder and Edward, 2011) to obtained clear reads (5GB). Trimmed sequenced were assembled with NOVOPlasty (Dierckxsens et al. 2017). Assembled sequences were annotated using DOGMA (Wyman et al. 2004) in accordance with the manual adjustment using BLAST (https://blast.ncbi.nlm.nih.gov/Blast.cgi), trNAscan-SE2.0 (Lowe and Chan 2016) was used to identify tRNA genes. Finally, the complete cp genome sequence of *P. tomentosa* was submitted to the Genbank with accession number MN548766.

The complete plastome genome of *P. tomentosa* is 164,213 bp in length with a circular topology. It has 37.3% GC content, a large single-copy (LSC) region of 81,102 bp and a small single-copy (SSC) region of 167,022 bp. It consists of 109 protein-coding genes, 44 tRNA genes, and 4 rRNA genes (*rrn4*, *rrn5*, *rrn16*, and *rrn23*).

For the purpose of phylogenetic analysis, in order to evaluate the phylogenetic position of *P. tomentosa*, among other *Asclepiadoideae*, seven species were downloaded from the Genbank database: *Biondia insignis* (MH748558), *Asclepias nivea* (NC022431), *Calotropis procera* (MH939982), *Cynanchum wilfordii* (KX352467), and two species from Rauvofloideae sub-family, members of Apocynaceae, were chosen as the outgroup: *Catharanthus roseus* (KC561139) and *Rhazya stricta* (KJ123753).

**Figure 1.**
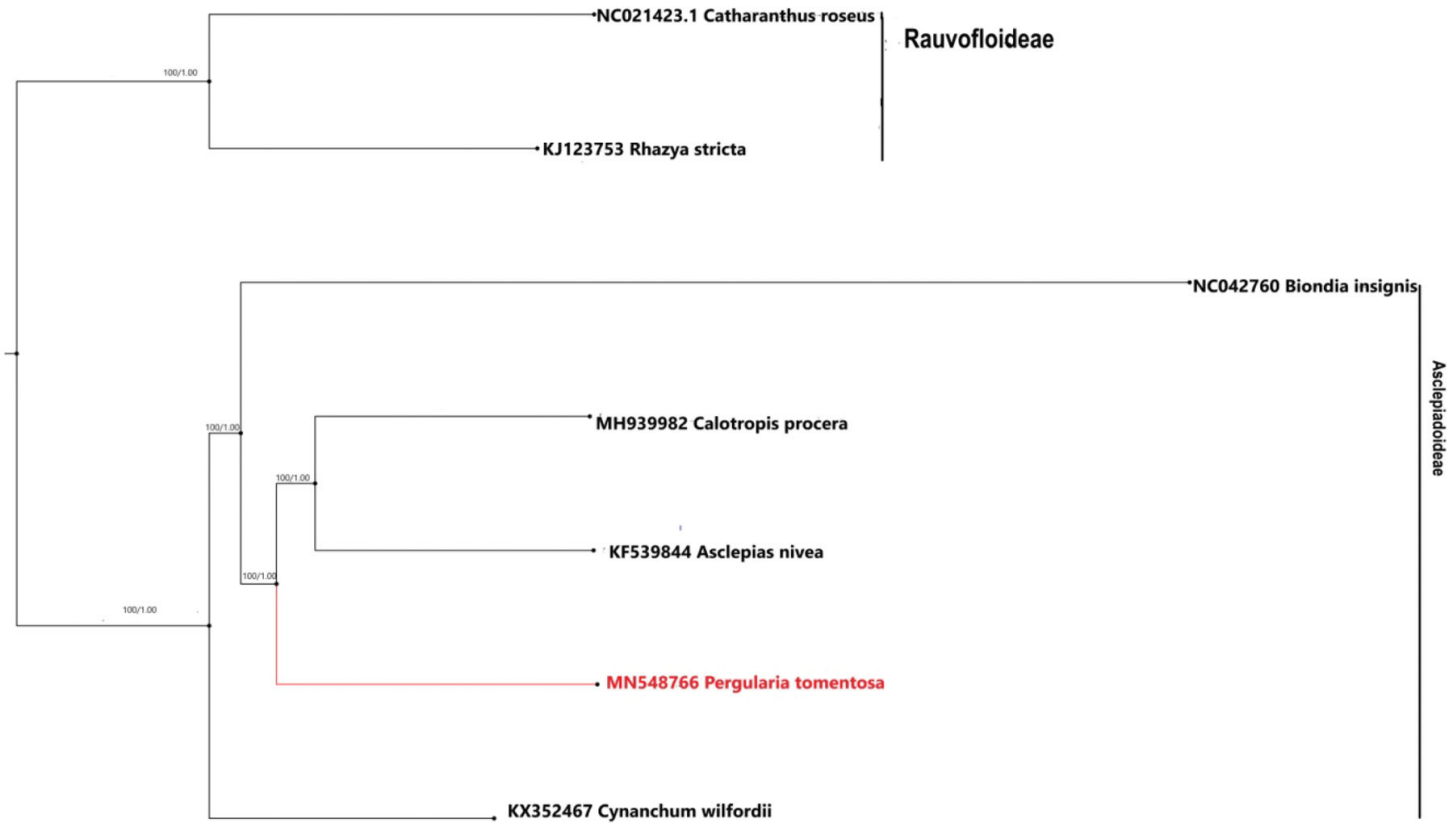
Bayesian Inference phylogenetic tree of *P. tomentosa* with other Asclepiadoideae species based on the complete chloroplast genome sequence. Numbers in the nodes represent posterior probability (PP) values.

The result of the phylogenetic analysis shows that *P. tomentosa* (MN548766) is correctly placed under the tribe Asclepiadeae together with other members of the clade, however, *C. procera* and *A. nivea* were from the same sub-tribe along with *P. tomentosa*, that is, Asclepiadeae, as indicated by the tree in Figure 1. The result of these studies can be used as bases for future evolutionary studies of Apocynaceae.
